# The microsporidian *Enterocytozoon hepatopenaei* is not the cause of white feces syndrome in whiteleg shrimp *Penaeus* (*Litopenaeus) vannamei*

**DOI:** 10.1186/1746-6148-9-139

**Published:** 2013-07-15

**Authors:** Amornrat Tangprasittipap, Jiraporn Srisala, Saisunee Chouwdee, Montagan Somboon, Niti Chuchird, Chalor Limsuwan, Thinnarat Srisuvan, Timothy W Flegel, Kallaya Sritunyalucksana

**Affiliations:** 1 Center of Excellence for Shrimp Molecular Biology and Biotechnology, Faculty of Science, Mahidol University, Rama VI rd, Bangkok 10400, Thailand; 2 Shrimp-Virus Interaction Laboratory, National Center for Genetic Engineering and Biotechnology, Soi Yothi, Rama VI rd, Bangkok 10400, Thailand; 3 National Center for Genetic Engineering and Biotechnology, National Science and Technology Development Agency (NSTDA), Thailand Science Park, Phaholyothin Road, Khlong Nueng, Khlong Luang, Pathum Thani 12120, Thailand; 4 Aquaculture Business Research Center, Kasetsart University, Bangkok 10900, Thailand; 5 Department of Biotechnology, Faculty of Science, Mahidol University, Rama VI rd, Bangkok 10400, Thailand; 6 Department of Livestock Development, 69/1 Phayathai Road, Bangkok 10400, Thailand

## Abstract

**Background:**

The microsporidian *Enterocytozoon hepatopenaei* was first described from Thailand in 2009 in farmed, indigenous giant tiger shrimp *Penaeus (Penaeus) monodon*. The natural reservoir for the parasite is still unknown. More recently, a microsporidian closely resembling it in morphology and tissue preference was found in Thai-farmed, exotic, whiteleg shrimp *Penaeus (Litopenaeus) vannamei* exhibiting white feces syndrome (WFS). Our objective was to compare the newly found pathogen with *E. hepatopenaei* and to determine its causal relationship with WFS.

**Results:**

Generic primers used to amplify a fragment of the small subunit ribosomal RNA (ssu rRNA) gene for cloning and sequencing revealed that the new parasite from WFS ponds had 99% sequence identity to that of *E. hepatopenaei*, suggesting it was conspecific. Normal histological analysis using tissue sections stained with hematoxylin and eosin (H&E) revealed that relatively few tubule epithelial cells exhibited spores, suggesting that the infections were light. However, the H&E results were deceptive since nested PCR and *in situ* hybridization analysis based on the cloned ssu rRNA gene fragment revealed very heavy infections in tubule epithelial cells in the central region of the hepatopancreas in the absence of spores. Despite these results, high prevalence of *E. hepatopenaei* in shrimp from ponds not exhibiting WFS and a pond that had recovered from WFS indicated no direct causal association between these infections and WFS. This was supported by laboratory oral challenge trials that revealed direct horizontal transmission to uninfected shrimp but no signs of WFS.

**Conclusions:**

The microsporidian newly found in *P. vannamei* is conspecific with previously described *E. hepatopenaei* and it is not causally associated with WFS. However, the deceptive severity of infections (much greater than previously reported in *P. monodon*) would undoubtedly have a negative effect on whiteleg shrimp growth and production efficiency and this could be exacerbated by the possibility of horizontal transmission revealed by laboratory challenge tests. Thus, it is recommended that the PCR and *in situ* hybridization methods developed herein be used to identify the natural reservoir species so they can be eliminated from the shrimp rearing system.

## Background

Several microsporidians have been reported as pathogens of penaeid shrimp [1]. Of these, two species have been reported to infect cultivated shrimp in Thailand. One of these is a species of *Agmasoma* previously called *Thelohania* that infects muscle tissue and connective tissue in the giant tiger shrimp *Penaeus (Penaeus) monodon* and the banana prawn *Penaeus (Feneropenaeus) merguiensis*[2-4]. It resembles morphologically the microsporidian *Agmasoma penaei* reported to infect *Penaeus (Litopenaeus) setiferus* and *Penaeus (Farfantepenaeus) duorarum*[1,5] in the Americas. More recently in Thailand it has been reported to also infect the same tissues in *Penaeus (Litopenaeus) vannamei*[6-8]. Spores from an unidentified microspridian have also been reported in muscles of *P. monodon* from Madagascar [9].

The other microsporidian reported from Thailand was a newly described species *Enterocytozoon hepatopenaei*[10] restricted to tubule epithelial cells of the hepatopancreas of *P. monodon*. In 2010, *E. hepatopenaei* was also reported from *P. monodon* exhibiting white feces syndrome (WFS) in Vietnam [11]. Here we report widespread infections of a microsporidian conspecific with *E. hepatopenaei* that was found in Thai-cultivated, exotic whiteleg shrimp *P. vannamei* exhibiting WFS. In addition, a nested PCR detection protocol is described together with its use in examining whiteleg shrimp from culture ponds and from oral challenge tests using hepatopancreatic tissue from shrimp with microsporidian infections.

## Results

### Small subunit ribosomal RNA gene fragment analysis

**Figure 1:**
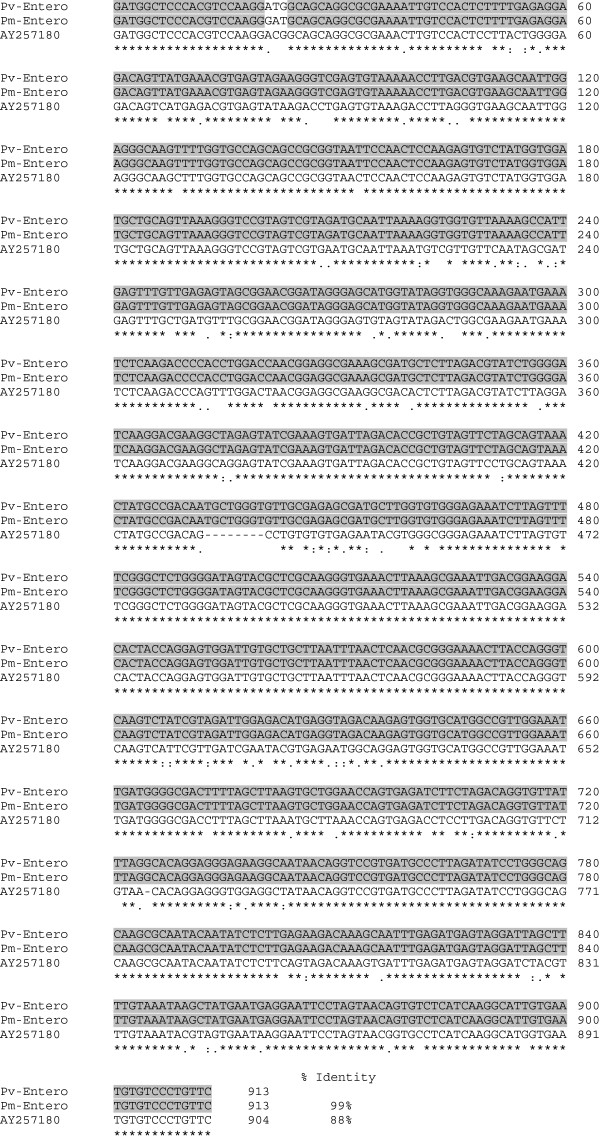
**Clustal-W alignment of microsporidian small subunit rRNA sequences.** The ssu rRNA sequence of *Enterocytozoon hepatopenaei* from *P. monodon* (Pm-Entero)(Genbank accession no. KF362129) and the microsporidian from *P. vannamei* (Pv-Entero) (Genbank accession no. KF362130) are compared with the matching region of the ssu rRNA gene of *E. bieneusi* (GenBank AY257180). Regions of 100% identity between Pv-Entero and Pm-Entero are outlined in grey background while 100% identity among all three species is indicated by asterisks.

When the MF primers designed from the ssu rRNA sequences of *Enterocytozoon* species [12] were used with template hepatopancreatic DNA extracts from *P. vannamei* infected with the microsporidian, a 951 bp amplicon was obtained (Additional file 1). This was within the range of the expected size of approximately 900–1000 bp, based on conserved regions of *Enterocytozoon* ssu rRNA sequences listed at GenBank (FJ496356) and the previous amplicon of 886 bp obtained from *P. monodon* infected with *Enterocytozoon hepatopenaei*[12]. Cloning and sequencing of 3 clones revealed 100% identical sequences for 2 clones and only 2 variable nucleotides for a third clone. A consensus sequence was concluded from the two identical clones and a 913 bp portion of the amplicon sequence (excluding the primer sequences, Genbank accession no. KF362130) was subjected to a general BLASTN search that yielded hits only for microsporidian sequence records. The top hits from the BLASTN search included *Enterocytozoon* isolated from *P. monodon* (GenBank FJ496356) at 96% identity, *Nucleospora salmonis* (GenBank U10883) at 89% identity and *E. bieneusi* (GenBank : AY257180) at 89% identity. The MF1 and MR1 primers were also used to amplify the ssu rRNA gene target from archived material of infected *P. monodon* used in previously published work [12] that gave rise to the GenBank record FJ496356. A consensus sequence was established from 3 clones also of 913 bp each and named Pm-Entero (Genbank accession no. KF362129). Clustal alignment of our 913 bp sequence from *P. vannamei* with our 913 bp sequence from *P. monodon* revealed 99% identity, indicating that infections arose from the same microsporidian species (Figure 1).

### PCR and *in situ* hybridization detection of the microsporidian in *P. vannamei*

With a preliminary field sample of 11 shrimp taken from one WFS pond and 10 from a nearby normal pond, PCR tests using primers specific for *E. hepatopenaei* (Table 1) revealed that 10/11 shrimp from the WFS pond were positive while all 10 from the normal pond were negative (Table 2). However, a later set of samples (Table 3) gave less clear-cut results, in that some ponds without signs of WFS gave a high prevalence (9/10 shrimp) positive for *E. hepatopenaei* by PCR (albeit mostly for the nested step), while other ponds with gross signs of WFS gave a low prevalence of PCR positive shrimp (4/10). In addition, one recovered pond with no signs of WFS (Table 4) had a high prevalence of shrimp (8/9) with extensive infections as determined by *in situ* hybridization. This constituted a poor correlation between gross signs of WFS and severity of *E. hepatopenaei* infection.

**Table 1:** Primer sequences used

**Primer name**	**Sequence (5′→ 3′)**	**Size of amplicon (bp)**
MF1	CCG GAG AGG GAG CCT GAGA	951
MR1	GAC GGG CGG TGT GTA CAA A	
**1**^**st **^**PCR step**		779
ENF779	CAG CAG GCG CGA AAA TTG TCC A	
ENR779	AAG AGA TAT TGT ATT GCG CTT GCT G	
**Nested PCR step**		176
ENF176	CAA CGC GGG AAA ACT TAC CA	
ENR176	ACC TGT TAT TGC CTT CTC CCT CC	
**DIG microsporidian probe**		411
ENF411	AGG TGG TGT TAA AAG CCA TTG AG	
ENR176	TAC CTC ATG TCT CCA ATC TAC GAT A	
**DIG GFP probe**		513
GFPF513	TTC ATC TGC ACC ACC GGC AAC CTG	
GFPR513	CTG GTA GTG GTC GGC GAG CTG CAC	

**Table 2:** PCR results from a preliminary sample of shrimp from a WFS pond and a normal pond nearby

	**WFS pond**		**Normal pond**	
**Sample #**	**1**^**st **^**PCR**	**Nested PCR**	**1**^**st **^**PCR**	**Nested PCR**
1	+	+	-	-
2	+	+	-	-
3	-	+	-	-
4	+	+	-	-
5	+	+	-	-
6	-	-	-	-
7	+	+	-	-
8	+	+	-	-
9	+	+	-	-
10	-	+	-	-
11	+	+		

**Table 3:** **PCR and *in situ *hybridization results for microsporidia in a second set of shrimp ponds**

**Normal shrimp**	**Pond 3 CV5 (n=10)**		***In situ***	**Pond 7 YOT4 (n =5)**		***In situ***
**sample number**	**1**^**st **^**PCR**	**2**^**nd **^**PCR**	**Hyb**	**1**^**st **^**PCR**	**2**^**nd **^**PCR**	**Hyb**
1	-	+	-	-	-	-
**2**	**-**	**-**	+	**-**	**-**	-
3	-	+	+	-	-	ND
4	+	+	++	-	-	ND
5	-	-	-	-	-	ND
6	-	+	-			
7	-	+	+			
8	-	-	+			
9	-	+	+			
10	-	+	+			
**Summary**	**Infected 9/10**			**Infected 0/5**		
**WFS shrimp**	**Pond 13 BAP (n=10)**		***In situ***	**Pond 6 YOT (n=10)**		***In situ***
**sample number**	**1**^**st **^**PCR**	**2**^**nd **^**PCR**	**Hyb**	**1**^**st **^**PCR**	**2**^**nd **^**PCR**	**Hyb**
1*	+	+	+	+	+	++
2	**-**	+	-	+	+	++
3*	-	-	-	+	+	ND
4	-	-	-	-	+	++
5	-	-	-	+	+	++
6	-	-	ND	+	+	ND
7*	-	-	ND	-	+	++
8*	-	-	ND	+	+	ND
9	-	+	-	-	-	ND
10	-	+	-	-	-	ND
**Summary**	**Infected 4/10**			**Infected 8/10**		

* Histology of shrimp in these ponds showed no signs of microsporidian infection but 4 showed signs of severe bacterial infections of the HP that may have masked any microsporidians present. The remaining 6 samples looked normal with no signs of microsporidian infection.

Detection of microsporidia by PCR and *in situ* hybridization in 2 normal and 2 WFS ponds. Hyb ++ indicates extensive positive *in situ* hybridization reactions in HP tissue while Hyb + indicates light focal positive reactions and Hyb – indicates no reaction. *ND* Not done.

**Table 4:** ***In situ *hybridization results of shrimp samples for a pond recovered from WFS**

**Recovered WFS shrimp pond**	
**Sample number**	***In situ *Hybridization**
1	++
2	++
3	-
4	++
5	++
6	++
7	++
8	++
9	++
**Summary**	**Infected 8/9**

Using the digoxygenin (DIG) labeled probe to confirm the validity of the PCR test, most (4/6) of the extensive *in situ* positive samples were 1^st^-step PCR positive (2 exceptions Table 3, Pond7 YOT4 & Pond6 YOT). Specimens that showed light positive *in situ* hybridization reactions (n = 7) were either 2^nd^-step (nested PCR) positive only (n = 4) or gave negative PCR reactions (n = 2), while one gave a 1^st^-step positive result (Pond 13 BAP-1). Of the 11 specimens that gave negative *in situ* hybridization test results, 6 gave negative PCR test results and 5 gave 2^nd^-step positive PCR results.

### Histopathology and *in situ* hybridization of infections in *P. vannamei*

**Figure 2:**
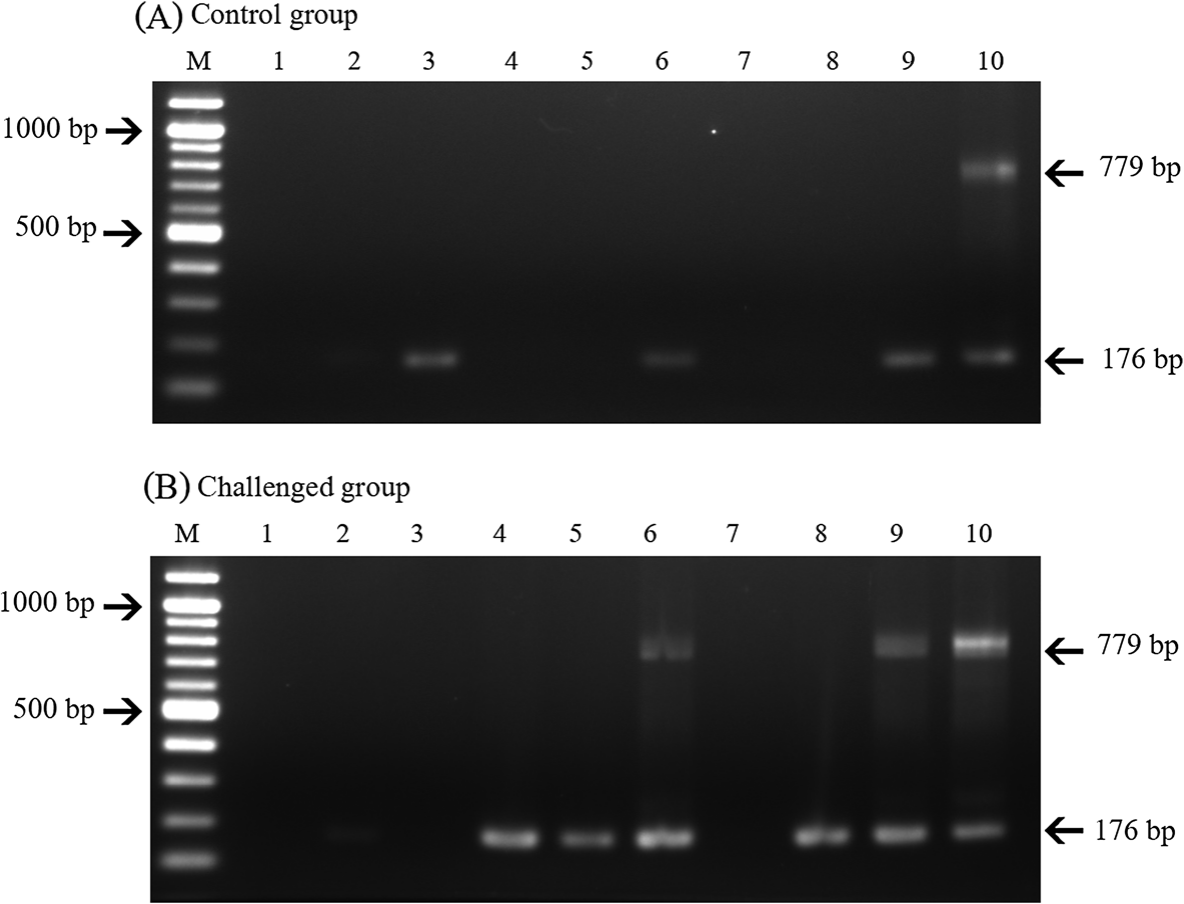
**Agarose gels showing nested PCR microsporidian-specific amplicons using 100 ng of total DNA template from hepatopancreatic tissue obtained from *P. vannamei *not challenged (A) or orally challenged (B) with the microsporidian.** Lane 1-3:Three specimens 2 days post-challenge; Lane 4-6: Three specimens 4 days post-challenge, Lane 7-9: Three specimens 7 days post-challenge; Lane 10: plasmid positive control. The amplicon sizes for the 1^st^ and 2^nd^-step PCR are 779 and 176 bp, respectively.

**Figure 3:**
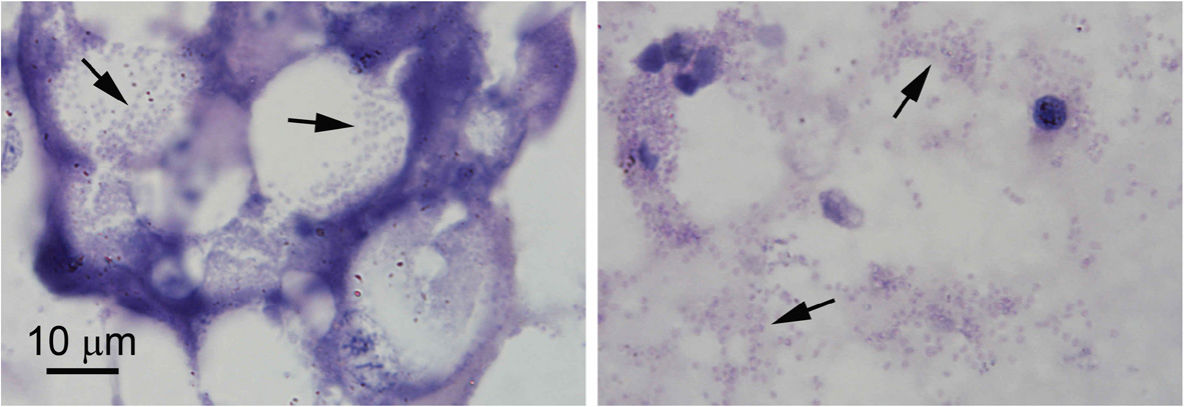
**Photomicrographs of microsporidian spores in hepatopancreatic cells.** Microsporidian spores (arrows) inside B cells of the hepatopancreatic tubule epithelium (left) and free in the tubule lumen (right). Such cells were present in the section in low numbers, giving a misleading impression of the extent of the severe microsporidian infection evident by in situ hybridization as seen in Figure 2. The magnification bar applies to both images.

As previously reported for infections of *E. hepatopenaei* in *P. monodon*, the number of hepatopancreatic cells showing spore formation in *P. vannamei* (Figure 2) was small, giving a superficial impression that the extent of the infections was very limited. The size of the spores (approximately 1 μm in length and less than 1 μm in width) and cytoplasmic location were also similar to those previously described for *E. hepatopenaei* in *P. monodon*. However, differences in *P. vannamei* included spore formation exclusively in B cells (Figure 2) and extensive infection of the medial and proximal tubule epithelial cells of the hepatopancreas in the absence of spores, as revealed by *in situ* hybridization (Figure 3). This was not the situation for previous reports on *E. hepatopenaei* in *P. monodon*, where relatively few cells produced spores or showed recognizable plasmodia, and only those cells were positive by *in situ* hybridization [12,13]. The cells giving positive *in situ* hybridization reactions in *P. vannamei* were restricted to the central region of the HP and did not extend to the distal region composed of E-cells. In the transitional zone between the medial and distal cells, pinpoint positive *in situ* hybridization reactions suggested that early infection stages occurred as the HP cells differentiated from E cells into B, F and R cells. Negative control slides using the GFP-DIG-labled probe gave no positive *in situ* hybridization reactions (not shown). At low and medium magnification by H&E staining (Figure 3a,c,e), there were no distinctive cytoplasmic features that revealed the microsporidian elements giving rise to positive *in situ* hybridization reactions in adjacent tissue sections. At the highest magnification by H&E staining (oil emersion lens), basophilic, cytoplasmic inclusions of highly variable, shape, size and number (Figure 3g) were present in the H&E stained cells and some of these may have been of microsporidian origin but they could not be unequivocally distinguished from other normal, basophilic cytoplasmic structures of the host. Because of this phenomenon, histopathological evaluation of the severity of these infections by H&E staining might be misleading, if the criterion used was the number of cells showing spores or other easily recognizable microsporidian structures.

### Laboratory challenge tests

**Figure 4:**
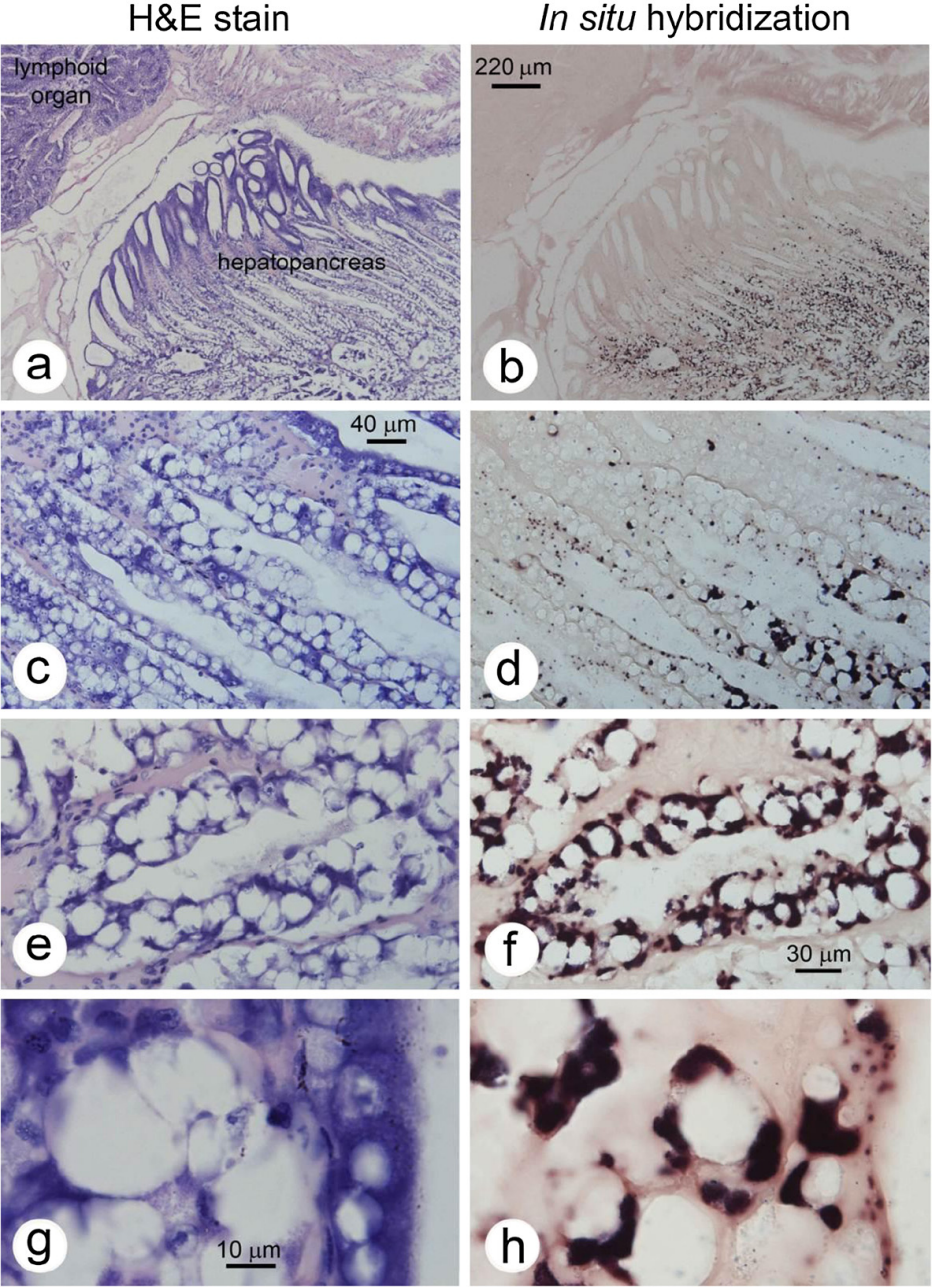
**Photomicrographs of infected hepatopancreatic tissue of *P. vannamei.*** The adjacent sections of shrimp tissue stained with H&E (column 1) and with the in situ hybridization probe (column 2) showing that hepatopancreatic cells of P. vannamei infected with the microsporidian cannot be easily detected by H&E staining even though extensive infection is revealed by in situ hybridization (dark brown to black staining). **(a/b)** Low magnification showing that positive reactions are restricted to the medial and proximal tubule epithelial cells of the shrimp hepatopancreas (HP) while the distal E cells are negative. Note that B cells dominate in the infected region. **(c/d)** Medium high magnification showing pinpoint positive, in situ hybridization reactions in the region of the HP adjacent to the distal E cells region. **(e/f)** High magnification clearly showing the difficulty in identifying infected cells by H&E staining but their clear revelation by in situ hybridization. **(g/h)** Very high magnification, emphasizing the features described in **(e/f)**.

Because of the association of *Enterocytozoon* infections with WFS in *P. vannamei* (this study) and *P. monodon*[11], a preliminary laboratory challenge test was carried out to determine the possibility of direct transmission using normal *P. vannamei* fed with hepatopancreatic tissue of shrimp that originated from a shrimp pond experiencing a WFS outbreak. Control shrimp were fed hepatopancreatic tissue from normal shrimp. Upon sampling the control shrimp on days 2, 4 and 7 after challenge, it was found that 1 in 3 sampled shrimp on each day gave weak microsporidian amplicons for the 2^nd^-step PCR (Figure 4), indicating that approximately 1/3 of the stock shrimp had already acquired light microsporidian infections before the experiment was initiated. Similarly, in the test group, one of the 3 sampled shrimp gave a weak 2^nd^-step microsporidian amplicon on day 2 after challenge, while stronger reactions were obtained on days 4 and 7, with two of these giving 1^st^-step positive results (Figure 4). None of the shrimp in either the test or control group showed gross signs of WFS or any mortality over the 7-day test period. These results indicated that *E. hepatopenaei* could be transmitted horizontally in *P. vannamei* by cannibalism, and this contrasted with *Agmasoma penaei* where transmission to shrimp is indirect from an alternate host [2-4].

## Discussion

Because of identical tissue specificity, identical spore size and a sequence identity of 99% obtained in comparison of ssu rRNA gene fragments of 913 bp, we concluded that the microsporidian newly found in *P. vannamei* was identical to *E. hepatopenaei* previously reported from *P. monodon*. However, we sequenced only 3 clones (98-99% identity) from each shrimp species to obtain the consensus sequences we used for phylogenetic comparison. Thus, according to veterinary sampling tables [14], could have missed sequences of other copies of the ssu rRNA gene that might have been present below the level of 64% prevalence in the template DNA and that might have differed from our consensus sequences by more than 1 or 2%. Thus, a more detailed genetic analysis of the whole, small and large subunit RNA genes, ITS regions and perhaps other genes would be needed to fully confirm our conclusion that *E. hepatopenaei* infects both of these shrimp species.

We have no explanation regarding the discrepancy between a strong 1^st^-step positive PCR test result despite the appearance of a light *in situ* hybridization reaction for one of the specimens from Pond 13 BAP. Of particular importance was a third set of samples from a recovered WFS pond where most of the shrimp (8/9) showed heavy *E. hepatopenaei* infections by *in situ* hybridization (Table 4) despite the absence of white feces. Unfortunately none of the HP samples of these latter shrimp were prepared for PCR. Overall, these results and those of the oral challenge test indicated an unlikely causal association between WSF and infection with *E. hepatopenaei*. On the other hand, it is possible that the severity of *E. hepatopenaei* infections could be increased by the unknown underlying causes of WFS, giving a superficial impression of causation.

The extensive nature of the *E. hepatopenaei* infections in many of the specimens examined suggests that there would be a high energy demand for the developing parasite and that this would have a negative effect on host growth. Given the difficulty in assessing the severity of infection by normal H&E staining, it would seem prudent to monitor ponds for *E. hepatopenaei* by PCR, especially if growth rates are lower than predicted in the absence of other more obvious causes. We have anecdotal information that one Thai farming operation has now adopted a policy of terminating and restocking culture ponds that show a high prevalence and severity of *E. hepatopenaei* infection indicated by one-step PCR positive reactions within the first month of cultivation, since their records show that these ponds exhibit uneconomic shrimp growth.

Our tests (not shown) have revealed that *E. hepatopenaei* is not present in the post larvae that originate from SPF stocks of *P. vannamei* and are used to stock cultivation ponds in Thailand. This indicates that infections found in the cultivation ponds occurred after the ponds were stocked, and it suggests that they resulted by transmission from a natural pond source such as an unknown local reservoir species. This contention is supported by the fact that *E. hepatopenaei* was discovered in Thailand in indigenous *P. monodon* long before it was found in exotic *P. vannamei*[10,13]. Thus, our results indicate that the best strategy for controlling its infections in cultivation ponds would be to focus on identification of the reservoir species and on eliminating them from the shrimp cultivation system. This approach has been successful for the control of *Agmasoma penaei* in cultivated shrimp in Thailand. It would also be worthwhile (because of the capability of horizontal transmission of *E. hepatopanaei*) to determine whether or not its residual spores in a disease outbreak pond are eliminated by current procedures for preparation of ponds between cultivation cycles.

## Conclusions

Although we have shown that the microsproidian *E. hepatopenaei* is often found in cultivated *P. vannemei* exhibiting WFS, it is unlikely that the parasite is causally associated with WFS, although the severity of *E. hepatopenaei* infections may be exacerbated by the underlying causes of WFS. Since this microsporidian is not present in Thai SPF stocks, pond infections are probably initiated by transmission from one or more local reservoir species. Thus, the most effective control strategy would be to identify the reservoir species and exclude it (them) from the shrimp production system. The PCR method and *in situ* hybridization method developed here will be useful in identifying reservoir species.

## Methods

### Sources of WFS and grossly normal farmed shrimp specimens

Since the Ethical Principles and Guidelines for the Use of Animals of the National Research Council of Thailand (1999) apply to vertebrates only and there is no official standard for invertebrates, we adapted its principles to shrimp. We also followed the guidelines of the Australian, New South Wales state government for the humane harvesting of fish and crustaceans (http://www.dpi.nsw.gov.au/agriculture/livestock/animal-welfare/general/fish/shellfish; 30 March 2013) with respect to details regarding the transport of the shrimp and their laboratory maintenance. With respect to processing the shrimp for histological analysis or for killing at the end of an experiment, the salt water/ice slurry method was used as recommended in the Australian guidelines.

One set of shrimp samples was collected from Surathani province in southern Thailand on 28 October 2010 and consisted of shrimp from 2 ponds exhibiting WFS, 1 pond recovered from WFS and 2 normal ponds. After stunning, the shrimp were swabbed with 70% ethanol and the carapace was removed so that approximately 100 mg of outer hepatopancreatic tissue (excluding contamination from the internal portion of the stomach and midgut) could be transferred aseptically to DNA extraction buffer (see below). The remainder of the whole hepatopancreas (including the anterior midgut caecum, the posterior chamber of the stomach and a portion of the midgut) was injected with Davidson’s fixative and processed for histological analysis as previously described [15]. A second set of juvenile *P. vannamei* specimens exhibiting white feces syndrome were collected from an intensive shrimp farm in Chanthaburi province, Thailand during August –September 2011 together with grossly normal shrimp from a nearby pond. Some of these shrimp were processed for DNA extraction from hepatopancreatic tissue as described above while the remaining were used in tests for transmission of the microsporidian by feeding of infected hepatopancreatic tissue to normal shrimp.

### Preparation of DNA templates

Hepatopancreatic tissue was homogenized in lysis buffer (50 mM Tris–HCl, pH 8.0, 50mM EDTA, 1% SDS, 10mM NaCl) containing 5 μg/ml proteinase K. Genomic DNA was isolated and purified by the phenol-chloroform method [16] and concentrations were determined by measuring UV absorption at 260 nm. All DNA templates were adjusted to a concentration of 50 ng/μl with distilled water for PCR tests.

### Cloning of a microsporidian small subunit ribosomal RNA gene fragment

A microsporidian ssu rRNA gene fragment was amplified from WFS *P. vannamei* by PCR as previously described [12]. Briefly, the primers MF1 and MR1 (Table 1) were designed from an ssu rRNA fragment of *Enterocytozoon hepatopenaei* isolated from *P. monodon* and relative to positions 242–260 and 1165–1183, respectively, of Genbank record FJ496356. The PCR process was carried out in a 25 μl reaction mixture containing PCR buffer, 0.2 mM dNTP, 1.5 mM MgCl_2_, 0.1 μM primers, 0.625 units Taq DNA polymerase (Invitrogen), and 1 μl template. The PCR protocol consisted of 35 cycles of denaturation at 94°C for 20 sec, annealing at 64°C for 20 sec, and extension at 72°C for 45 sec, followed by a 5 min final extension at 72°C. An srRNA gene fragment of 900–1,000 bp was amplified and cloned using a pGEMT-easy cloning Kit (Promega). Plasmids were extracted from three clones, purified using a plasmid extract kit (Geneaid) and sequenced in both directions by two universal vector primers, SP6 and T7, by Macrogen, Korea. The consensus sequence obtained (minus the primers) was subjected to a BLASTN (http://www.ncbi.nlm.nih.gov/BLAST) search against the GenBank database and then it was aligned with the corresponding region of the ssu rRNA sequence of *E. hepatopenaei* (Genbank : FJ496356) using Clustal-W2 (http://www.ebi.ac.uk/Tools/msa/clustalw2/). Among three clone sequences, two sequences were 100% identical, whereas one was different at 2 /951 bases analyzed. The clone named MF12 was used as the positive control template for PCR reactions.

To verify the GenBank record FJ496356 for the ssu rRNA sequence of the microsporidian *E. hepatopenaei* from *P. monodon*[12], we used the same PCR protocol described above to re-amplify and re-clone the ssu rRNA gene fragment from archived DNA used as the template that gave rise to the FJ496356 record. The amplicon was cloned and 3 clones were sequenced to obtain a consensus sequence.

### PCR detection of the microsporidian infection in *P. vannamei*

Two pairs of specific primers, ENF779/ ENR779 and ENF176/ENR176 were designed from the amplicon described above for a nested PCR protocol to enhance specificity and sensitivity for detection of the new microsporidian (Table 1). The PCR amplification was carried out in 25 μl reaction mixture containing, 200 mM dNTP, 1.5 mM MgCl_2_, 0.1 mM primers and 0.625 unit Taq polymerase (Invitrogen). For the first step PCR reaction, the protocol consisted of initial denaturation at 94°C for 3 min followed by 35 cycles of denaturation at 94°C for 20 sec, annealing at 58°C for 20 sec and extension at 72°C for 45 sec with a final extension at 72°C for 5 min. The second, nested PCR reaction was carried out using 1 μl of the first PCR product as template. The PCR reaction conditions consisted of initial denaturation at 94°C for 3 min followed by 35 cycles of denaturation at 94°C for 20 sec, annealing at 64°C for 20 sec and extension at 72°C for 20 sec with an additional extension at 72°C for 5 min. The PCR products were visualized by 1.5% of agarose gel electrophoresis, ethidium bromide staining and gel placement on a UV transillumator. To determine the sensitivity of detection, 10-fold serial dilutions (10^6^ – 1 copies of plasmid) were prepared and subjected to the nested PCR protocol.

### *In situ* hybridization

A Dig-PCR labeling Kit (Roche, Germany) was used to prepare a probe for *in situ* hybridization using the primers shown in Table 1. A similarly labeled GFP-Dig probe was used as a negative control. Plasmid clones MF12 and pEGFP–N1 (Clontech) containing relevant inserts were used as templates for *Enterocytozoon* sp. and the negative control, respectively. Dig-labeled probes were purified using a PCR purification kit (Geneaid) and labeling efficiency was determined by dot blot hybridization. Shrimp were fixed in Davidson's fixative overnight before processing for routine paraffin embedding [15]. Tissue sections were digested with 10 μg/ml Proteinase K (Invitrogen, USA) in TNE buffer for 10 min at 37°C. Each section was overlaid with 200 μl of pre-hybridization solution [4 × SSC and 50% (v/v) deionized formamide] and incubated at 37°C for 30 min before the solution was replaced with 200 μl of hybridization mix containing the DIG-labeled probe (approximately 20 ng/ slide) and covered with a coverslip. The hybridization reaction was carried out at 42°C for 20 h in a humid chamber to avoid evaporation. After the sections were washed with high stringency, they were incubated with 0.5% blocking solution (Roche, Germany) for 30 min at room termperature. The sections were incubated with alkaline phosphatase-conjugated anti-digoxigenin antibody (1:500 dilution). Unbound antibody was washed off twice and equilibrated in detection buffer (100 mM Tris–HCl, 100 mM NaCl and 50 mM MgCl_2_, pH 9.5). The signal was developed by addition of NBT-BCIP substrate (Roche, Germany) and counterstaining was accomplished with Bismarck brown Y (Sigma, USA). The slides were observed and photographed using an Olympus microscope with a digital camera.

### Laboratory challenge tests

Pacific white shrimp weighing 6–8 grams were obtained from a farm in Chanthaburi province, Thailand, and acclimated at the Aquaculture Business Research Center Laboratory, Faculty of Fisheries, Kasetsart University for 1 week. During the acclimatization period, shrimp were fed with commercial pellet feed. After that, 60 shrimp were randomly stocked in 6 aquaria (80 l each) with 10 shrimp per aquarium. The shrimp were divided into 2 groups of 30 animals (i.e., 3 aquaria) per group. Shrimp from the treatment group were fed with hepatopancreatic tissue of *E. hepatopenaei*-infected shrimp every day for 7 days (once a day and another meal fed with commercial pellet feed) while shrimp from the control group were fed with a commercial pellet feed twice a day. Dissolved oxygen (DO), Salinity, pH, and temperature during the acclimation period and the experiment were maintained at 4 ppm, 25 ppt, 7.8–8.0, and 28°C, respectively. The test was terminated after 7 days. The shrimp were sampled (one from each aquarium) on days 2, 4 and 7 after the feeding for PCR testing using the *E. hepatopenaei* primers. They were also monitored for mortality and signs of WFS.

## Competing interests

The authors declare that they have no competing interests.

## Authors’ contributions

AT carried out the in situ hybridization studies, histological examination and drafted the manuscript. SC carried out the histological examination. JS carried out the molecular cloning and sequence analysis. MS, NC and CL participated in the experimental designs, performed bioassays and specimen collection. TS participated in design of the study and preparation of the specimens. TF and KS participated in the design of the study and coordination and completed the manuscript. All authors read and approved the final manuscript.

## Supplementary Material

Additional file 1 **Agarose gel analysis of PCR amplicons obtained using MF primers from **[12]**.** Lane 1: MF12 plasmid positive control template; Lane 2: Total DNA extract template from a heavily infected shrimp; Lane 3: Same as lane 2 but lightly infected; Lane 4: Negative control DNA template from an uninfected shrimp; Lane 5: Distilled water template negative control. Click here for file
